# SARS-CoV-2 RNA Testing Using Different Assays—Impact on Testing Strategies in a Clinical Setting

**DOI:** 10.3390/ijms232112845

**Published:** 2022-10-25

**Authors:** Gerald M. Eibinger, Harald H. Kessler, Evelyn Stelzl, Klaus Vander, Anita Weber-Lassacher, Wilfried Renner, Markus Herrmann

**Affiliations:** 1 Clinical Institute of Medical and Chemical Laboratory Diagnostics, Medical University of Graz, 8036 Graz, Austria; 2 Institute of Hygiene, Microbiology and Environmental Medicine, Medical University of Graz, 8036 Graz, Austria; 3 Institute of Hospital Hygiene and Microbiology, University Hospital Graz, 8036 Graz, Austria

**Keywords:** SARS-CoV-2, COVID-19, RT-qPCR, Ct value, testing strategy

## Abstract

In order to assess SARS-CoV-2 real time quantitative polymerase chain reaction (RT-qPCR) results in a real-life setting, three independent laboratories in Graz (Austria) set up a continuous cross comparison schedule. The following test systems were used: The QIAGEN NeuMoDx SARS-CoV-2 Assay, the Allplex™ 2019-nCoV Assay (Seegene) on a MicroLab Nimbus (Hamilton) platform combined with RealStar SARS-CoV-2 RT-PCR Assay (Altona Diagnostics GmbH), and the cobas SARS-CoV-2 test on a fully automated cobas 6800 system (Roche). A total of 200 samples were analysed, 184 (92%) were found to be concordant with all testing platforms, 14 (7%) discordant. Two (1%) samples tested invalid on a single platform and were excluded from further analysis. Discordant results were distributed randomly across the assays. The Ct values from all assays correlated closely with each other. All discordant samples showed Ct values ≥ 26. SARS-CoV-2 RT-qPCR assays may show considerable variability, especially in samples with low viral RNA concentrations. Decision makers should thus balance the advantages and disadvantages of RT-qPCR for mass screening and adopt suitable strategies that ensure a rational management of positive samples with high Ct values.

## 1. Introduction

SARS-CoV-2 testing by real time quantitative polymerase chain reaction (RT-qPCR) has been pivotal for patients, health care providers, and political decision makers to manage the ongoing COVID-19 pandemic. Due to its high sensitivity and specificity, SARS-CoV-2 RT-qPCR testing allows the early identification of infected individuals regardless of clinical symptoms. This aspect is of particular importance, as infected individuals are already contagious during the incubation period, and long before the development of SARS-CoV-2 specific antibodies [1]. Because of its superior analytical performance, SARS-CoV-2 RT-qPCR testing has become the preferred diagnostic tool for clinical decision-making, quarantine measures, and epidemiologic issues [1,2]. As a result, medical laboratories were facing an unprecedented escalation of SARS-CoV-2 RT-qPCR requests within a few months. Austria, an early adopter of an RT-qPCR based containment strategy, has performed almost 200,000,000 tests [3]. These figures illustrate the unique situation that laboratories were facing during the past two years. A major challenge, especially in the early phase of the pandemic, was the limited availability of consumables, reagents, and instruments. It took diagnostic manufacturers more than one year to scale up their production to a level that satisfied market requirements. As a result, most laboratories had to purchase multiple systems in order to ensure continuity of their operation. Public decision makers, healthcare providers, and patients were not aware of the inherent limitations of laboratory tests. Especially, patients, colleagues, and public authorities often challenged unexpected positive results in asymptomatic individuals that were not confirmed upon retesting in another laboratory. In summary, this has raised concerns about the analytical quality of laboratories.

Using a highly sensitive method, such as RT-qPCR, for large scale SARS-CoV-2 testing is particularly critical as minimal amounts of viral RNA can produce a positive result. In fact, some CE-IVD certified SARS-CoV-2 RT-qPCR tests run up to 45 cycles in order to maximize sensitivity. However, in vitro experiments suggest that cycle threshold (Ct) values of 26 and higher indicate a low viral load, which is insufficient to infect cultured cells [4]. Despite the negligible epidemiologic risk associated with Ct values above 26, such results are reported as positive.

In order to assess SARS-CoV-2 RT-qPCR results in a real-life setting, three independent laboratories in the city of Graz (Austria) set up a quality assurance initiative establishing a continuous cross comparison scheme, where samples were exchanged daily for several months. The participating laboratories were all involved in routine SARS-CoV-2 RT-qPCR testing throughout the pandemic and used different analytical systems.

## 2. Results

**Figure 1:**
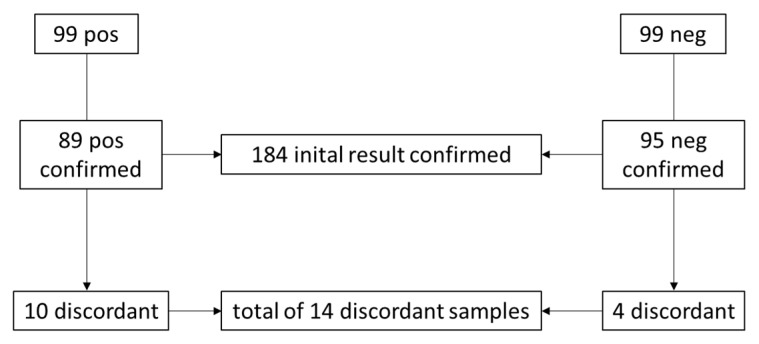
Concordance analysis of qualitative results in 99 positive and 99 negative samples of the inter-laboratory comparison. Samples were classified as discordant if at least one of the comparison assays did not confirm the initial result obtained with the QIAGEN test.

A total of 200 samples were tested with three different testing platforms (Table 1). Two samples (1%) had to be excluded from further analysis due to invalid testing results. Based on the QIAGEN results, 99 samples were SARS-CoV-2 positive with Ct values ranging from 12 to 39, and 99 samples were found to be negative. Of 198 samples included in further analysis, 184 (92.9%) were found to be concordant with all three assays, while 14 (7.1%) were found to be discordant. Eighty-nine (89.9%) of the 99 initially positive results were found to be positive and 95 (96.0%) of the initially negative results were found to be negative with both alternative assays (Figure 1).

**Figure 2:**
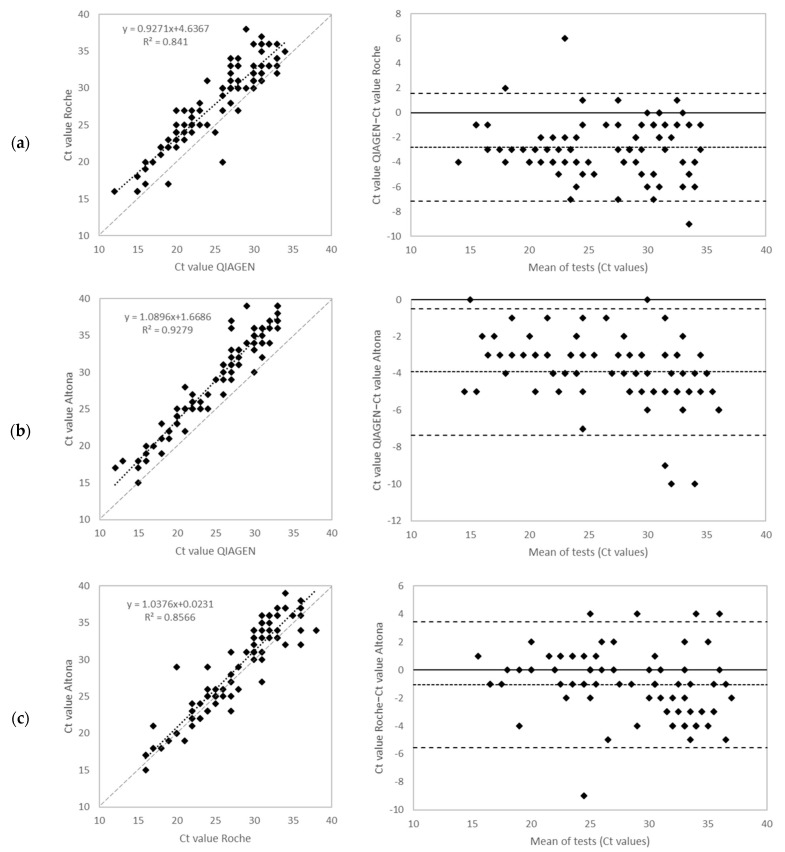
Comparison of Ct values obtained with QIAGEN and Roche (**a**), QIAGEN and Altona (**b**), Roche and Altona (**c**).

For all concordant results, Ct values obtained were compared (Figure 2). Despite good agreement of the qualitative results, the QIAGEN method tended to yield lower Ct values compared to Roche and Altona. Amongst the 89 samples that were positive with all three methods, results obtained from 77 (86.5%) samples showed differences of 5 cycles or less. The results obtained from 12 (13.5%) samples differed by 6 to 10 cycles. Variations of more than 10 cycles where not observed.

All discordant samples are shown in Table 2. No obvious preponderance of the initial QIAGEN result as being positive or negative was observed. Only two samples that had been found to be negative with the initial QIAGEN test tested positive with both of the other methods, showing Ct values ≥ 36.

## 3. Discussion

Here we show that the parallel use of different SARS-CoV-2 RT-qPCR systems, which was often necessary during the COVID-19 pandemic, harbors a significant risk of discordant or non-reproducible results, especially when testing low risk populations. Importantly, discordant results were obtained with all assays included. The potential implications of variable results especially in samples with low amounts of viral RNA should motivate decision makers to adapt existing RT-qPCR based screening programs for SARS-CoV-2, especially in asymptomatic individuals.

In this study, the SARS-CoV-2 RT-qPCR assays from QIAGEN, Roche, and Altona agreed reasonably well. Recently published data from the National Austrian External Quality Assurance (EQA) scheme showed that 1–7% of the recorded results for individual samples included in a proficiency panel were reported false negative [5]. The highest false negative rate was seen in a sample with a Ct value of 36 in that program. In contrast, there were no false positive results. Taking into account that 109 laboratories with a total of 134 individual test systems participated in this program, the results can be regarded as rather robust. However, as there were only seven samples distributed to the participants, heterogeneity of the sample matrix was limited. In clinical practice, the characteristics of individual swab samples can differ widely, which represents a relevant source of error that is not sufficiently captured by EQA programs. For example, swab samples can be rather liquid with no solid components contained, or viscous with mucous aggregates, inflammatory cells, and other solid particles. In contrast to the analysis of EQA data with only a few well characterized samples, the present study was focused on the comparability of results in a real-life setting with a broad range of variables that can influence the analysis, including sample input volume and nucleic acid extraction efficacy. Moreover, manual errors may occur when using semi-automated tests. Another study from Buchta et al. investigated the variability of Ct values in EQA samples [6]. Three positive and two negative samples were analysed by 66 laboratories with 92 different protocols. While the mean Ct values for the E-, N-, S-, RdRp-, and ORF1ab genes varied by less than two cycles, nearly 8% of the reported results differed by four or more cycles. The maximum deviation from the respective mean was 18 cycles. However, the agreement of Ct values obtained with the same method, but in different laboratories, was very good. Substantial variability of SARS-CoV-2 RT-qPCR results is further supported by a study from Malecki M et al. [7], that analyzed the variation of Ct values in EQA samples using different extraction protocols, PCR instruments, and reagent kits. A maximum difference of nine cycles was recorded, and in one weakly positive sample, 3 out of 12 analytical systems yielded false negative results. Based on these results, the authors concluded that laboratories should carefully validate the methods that they use and adapt the interpretation of results accordingly. It has to be emphasized that EQA data should be interpreted with caution as the number of samples is limited and the sample matrix is usually optimized. In clinical practice, more influencing factors are routinely encountered and the present results suggest that variability is expected to be even higher.

While the issue of false negative SARS-CoV-2 RT-qPCR results has been discussed widely in the scientific community, much less is known about false positives [8,9]. Without sufficient clinical information, it is almost impossible for laboratories to recognize false positive results. In cases with an elevated pre-test probability, such as symptomatic individuals or persons who had contact with actual or suspected carriers of the virus, public authorities recommend regarding positive results as true [10]. However, in asymptomatic subjects without a specific risk of infection, positive RT-qPCR tests are unexpected and should raise suspicion. Braunstein et al. analyzed the false positive rate in the staff screening program of the Walt Disney Company. From more than 120,000 tests performed, 239 positive tests were further investigated because of previously negative results [8]. Retesting at two occasions after at least 24 h confirmed 54 (22.6%) of these results as false positives. In such a setting, the diagnostic accuracy of SARS-CoV-2 RT-qPCR tests is expected to be lower. In fact, the positive predictive value was only 77.4%. Unfortunately, the authors did not specify the method that was used for SARS-CoV-2 RT-qPCR testing. 

Causes of false positives SARS-CoV-2 RT-qPCR tests include contamination during sampling, RNA extraction, and PCR amplification [8,11]. Therefore, trained personnel should perform sample collection in adherence with appropriate protocols. Workbenches and technical equipment, such as pipettes, containment hoods, or instruments, should be thoroughly cleaned and regularly checked for contamination. Reagents and other consumables can also be contaminated during the production process [12,13,14]. Finally, false positive results can also occur due to sample mix-ups, data entry or transmission errors, non-specific reactions, and inappropriate reporting of indeterminate results as positive [8]. 

The present results provide clear evidence that SARS-CoV-2 RT-qPCR results vary when viral load is low. We and others have shown that individuals with low viral load are usually not infectious, as shown by cell culture-based infection assays [4,15]. Therefore, in low-risk populations, SARS-CoV-2 positive individuals with high Ct values should be isolated until retesting after 24–48 h. This strategy would help to separate false positives from true positives with low viral load, such as in the very early phase of an infection. An alternative approach is screening with a thoroughly validated rapid antigen test. As shown by Kessler et al., the rapid antigen test from Roche Diagnostics can reliably differentiate between infectious and non-infectious individuals [4]. Despite a lower sensitivity, this test offers satisfactory specificity and is unlikely to miss a clinically relevant SARS-CoV-2 infection. Pickering et al. reported similar findings for other widely used rapid antigen tests [16].

A major strength of the present study is that the initial analysis by QIAGEN and retesting with another assay were performed using the same sample, which rules out sample collection errors. In addition, blinded retesting was usually performed within 24 h, but not later than 72 h (on weekends). Moreover, the tested samples were rather diverse as they were derived from symptomatic patients as well as from asymptomatic hospital employees. This ensures that the results account for a broad range of variables that are encountered in real life. Without clinical information and the possibility to recollect additional samples, a further characterization of discordant results was impossible in this study. Discordant results can occur for various reasons including use of different target genes, different nucleic acid extraction and amplification efficiencies, and the number of manipulations involved in sample processing [17]. Despite rapid retesting of all samples, some degree of RNA degradation in individual samples cannot be ruled out completely. However, own stability experiments have shown that short-term storage at 4–8 °C does not cause a shift of Ct values. Given that in the present study most Ct values agreed within five cycles, significant sample degradation can be largely excluded.

In conclusion, commercial SARS-CoV-2 RT-qPCR assays show considerable variability, which has an important impact on results and may lead to inappropriate isolation decisions. Therefore, decision makers should balance the advantages and disadvantages of RT-qPCR for mass screening and adopt suitable strategies that ensure a rational management of weakly positive results. Alternatively, rapid antigen testing may be considered as a cost-effective alternative to identify infectious individuals in low-risk populations.

## 4. Materials and Methods

### 4.1. Study Design

The Clinical Institute of Medical and Chemical Laboratory Diagnostics (CIMCL) at the Medical University of Graz (MUG, Graz, Austria) selected daily two swab samples for SARS-CoV-2 RT-qPCR cross-comparison with two other laboratories. One sample was SARS-CoV-2 positive and the other one negative based on the QIAGEN NeuMoDx SARS-CoV-2 Assay (QIAGEN, Hilden, Germany; see below). Oropharyngeal or nasopharyngeal swabs were collected using the Copan eSwab™ (Copan, Brescia, Italy) collection and transport system. From each sample, two aliquots were produced. One aliquot was sent to the Diagnostic and Research Institute of Hygiene, Microbiology, and Environmental Medicine (IHMU) at the MUG, the other one to the Institute of Hospital Hygiene and Microbiology (IKM) at the University Hospital Graz, Austria. Both laboratories were blinded to the primary results produced by the CIMCL. All methods used are CE-IVD labeled.

### 4.2. SARS-CoV-2 RT-qPCR Testing

All molecular tests used in this study were performed according to the manufacturers’ protocols. Characteristics of tests are shown in Table 1. All samples were initially tested with the QIAGEN NeuMoDx SARS-CoV-2 Assay on a QIAGEN NeuMoDx 288 fully automated molecular analyzer (QIAGEN, Hilden, Germany) at the CIMCL. The CIMCL performs daily quality control measurements and participates successfully in an external quality assurance program (ÖQUASTA, Wien, Austria). The IHMU analyzed the samples with the cobas SARS-CoV-2 test (Roche Molecular Diagnostics, Pleasanton, CA, USA) on a fully automated cobas 6800 system (Roche Molecular Diagnostics, Rotkreuz, Switzerland). The IHMU performs within-run quality control measurements and participates successfully in an external quality assurance program (QCMD, Glasgow, Scotland; https://www.qcmd.org/, accessed on 20 September 2022). The IKM tested the samples with a semi-automated RT-qPCR method. Viral RNA was extracted from 200 μL of sample with the StarMag viral DNA/RNA 200 C Kit (Seegene, Seoul, South Korea) on a MicroLab Nimbus (Hamilton Company, Reno, NV, USA) automated extraction platform. Extracted RNA was eluted in 100 µL of buffer. Then, 10 µL of the eluate was used for SARS-CoV-2 amplification and detection with the RealStar SARS CoV-2 RT-PCR Assay (Altona Diagnostics GmbH, Hamburg, Germany). Amplification and detection were performed on a Light Cycler 480 real-time thermal cycler (Roche Molecular Diagnostics, Penzberg, Germany). The IKM laboratory performs daily quality control measurements and participates successfully in an external quality assurance program (Instand e.V., Düsseldorf, Germany; https://www.instand-ev.de, accessed on 20 September 2022).

### 4.3. Statistics

Qualitative results and Ct values were recorded in a Microsoft Excel data base. First, we analyzed the qualitative agreement of all methods by plotting the number of positive and negative results for each method with the initial analysis as reference. The qualitative results of the NeuMoDx 288 were compared with those of the other methods by chi-square test for categorical variables. Next, we identified samples with discordant results, where at least one method produced a discordant result compared to the initial analysis. With SARS-CoV-2 RT-qPCR, qualitative results are generated; however, many countries obliged laboratories to report Ct values as an estimate of viral load. Therefore, positive results were used to perform Spearman correlation analyses between the Ct values of the NeuMoDx 288 method and each of the other methods. The Ct value differences were rounded to whole numbers. Samples with at least one negative result were classified as discordant. Statistical calculations were performed with SPSS Statistics 28.0.1 (IBM Corp., Armonk, NY, USA).

## Figures and Tables

**Table 1:** Characteristics of all tests used in this study.

Manufacturer	QIAGEN	Roche	Altona
Test name	NeuMoDx SARS-CoV-2 Assay	cobas SARS-CoV-2 test	Allplex RealStar
Instruments	NeuMoDx	Cobas 6800	MicroLab Nimbus, LC 480
Target genes	NSP2, N	ORF1a/b, E	E, S
Automation	Fully automated	Fully automated	Partly automated
Study site	CIMCL	IHMU	IKM

**Table 2:** Results of discordant samples (numbers represent Ct values obtained by different tests).

Sample No.	QIAGEN	Roche	Altona
1	26	neg	30
2	28	33	neg
3	29	neg	39
4	30	33	neg
5	31	37	neg
6	32	36	neg
7	33	neg	39
8	33	neg	39
9	33	neg	neg
10	34	35	neg
11	neg	36	38
12	neg	38	36
13	neg	neg	37
14	neg	neg	39

## Data Availability

Not applicable.
